# Super-enhancer omics in stem cell

**DOI:** 10.1186/s12943-024-02066-z

**Published:** 2024-08-01

**Authors:** Hongying Ma, Jian Qu, Zicheng Pang, Jian Luo, Min Yan, Weixin Xu, Haihui Zhuang, Linxin Liu, Qiang Qu

**Affiliations:** 1 Department of Pharmacy, Xiangya Hospital, Central South University, No.87 Xiangya Road, Changsha, 410008 People’s Republic of China; 2 Institute for Rational and Safe Medication Practices, National Clinical Research Center for Geriatric Disorders, Xiangya Hospital, Central South University, Changsha, 410008 People’s Republic of China; 3 Department of Pharmacy, the Second Xiangya Hospital, Institute of Clinical Pharmacy, Central South University, Changsha, 410011 People’s Republic of China; 4 Hunan key laboratory of the Research and Development of Novel Pharmaceutical Preparations, Changsha Medical University, Changsha, 410219 China; 5 Department of Medical Genetics, School of Basic Medical Sciences, Southern Medical University, Guangzhou, 510515 China; 6 Department of Hematology, Xiangya Hospital, Central South University, Changsha, 410011 People’s Republic of China

**Keywords:** Super-enhancer omics, Multi-omics, Transcription, Stem cell, Cancer stem cell

## Abstract

The hallmarks of stem cells, such as proliferation, self-renewal, development, differentiation, and regeneration, are critical to maintain stem cell identity which is sustained by genetic and epigenetic factors. Super-enhancers (SEs), which consist of clusters of active enhancers, play a central role in maintaining stemness hallmarks by specifically transcriptional model. The SE-navigated transcriptional complex, including SEs, non-coding RNAs, master transcriptional factors, Mediators and other co-activators, forms phase-separated condensates, which offers a toggle for directing diverse stem cell fate. With the burgeoning technologies of multiple-omics applied to examine different aspects of SE, we firstly raise the concept of “super-enhancer omics”, inextricably linking to Pan-omics. In the review, we discuss the spatiotemporal organization and concepts of SEs, and describe links between SE-navigated transcriptional complex and stem cell features, such as stem cell identity, self-renewal, pluripotency, differentiation and development. We also elucidate the mechanism of stemness and oncogenic SEs modulating cancer stem cells via genomic and epigenetic alterations hijack in cancer stem cell. Additionally, we discuss the potential of targeting components of the SE complex using small molecule compounds, genome editing, and antisense oligonucleotides to treat SE-associated organ dysfunction and diseases, including cancer. This review also provides insights into the future of stem cell research through the paradigm of SEs.

## Introduction

At present, scientists have reached a consensus that human stem cells obtained from embryos, fetuses, or adults possess distinct characteristics known as “stemness hallmarks”, enabling them to undergo differentiation into various cell types, given specific conditions. These “hallmarks”, including proliferation, self-renewal, development, differentiation and regeneration, are critical for the identity of stem cell [1, 2]. However, our incomplete understanding of how the regulatory networks govern stemness hallmarks impedes the widespread implementation of stem-cell-derived therapeutics.

With the completion of the human genome project in 2003 and the mapping of the epigenome in 2015, the post-genome era has provided an opportunity to comprehensively understand the genome and epigenome [3]. However, the upstream transcriptional machinery controlled by the genome and epigenome remains largely unknown. Enhancers, a short cis-regulatory element (50–1500 bp) in the genome, which were experimentally demonstrated over 35 years ago [4], determine the specification of cell identity by the dynamic communication of enhancer-promoter [5]. Mechanisms of facilitating three-dimensional (3D) enhancer-promoter communication and multi-connected hubs have been proposed [5], including the extrusion of DNA loops by the Cohesin complex, the Mediator complex interacting with both transcriptional factors (TFs) and the RNA Pol II pre-initiation complex (PIC), and the interplay between TFs clustering and phase separation.

**Fig. 1:**
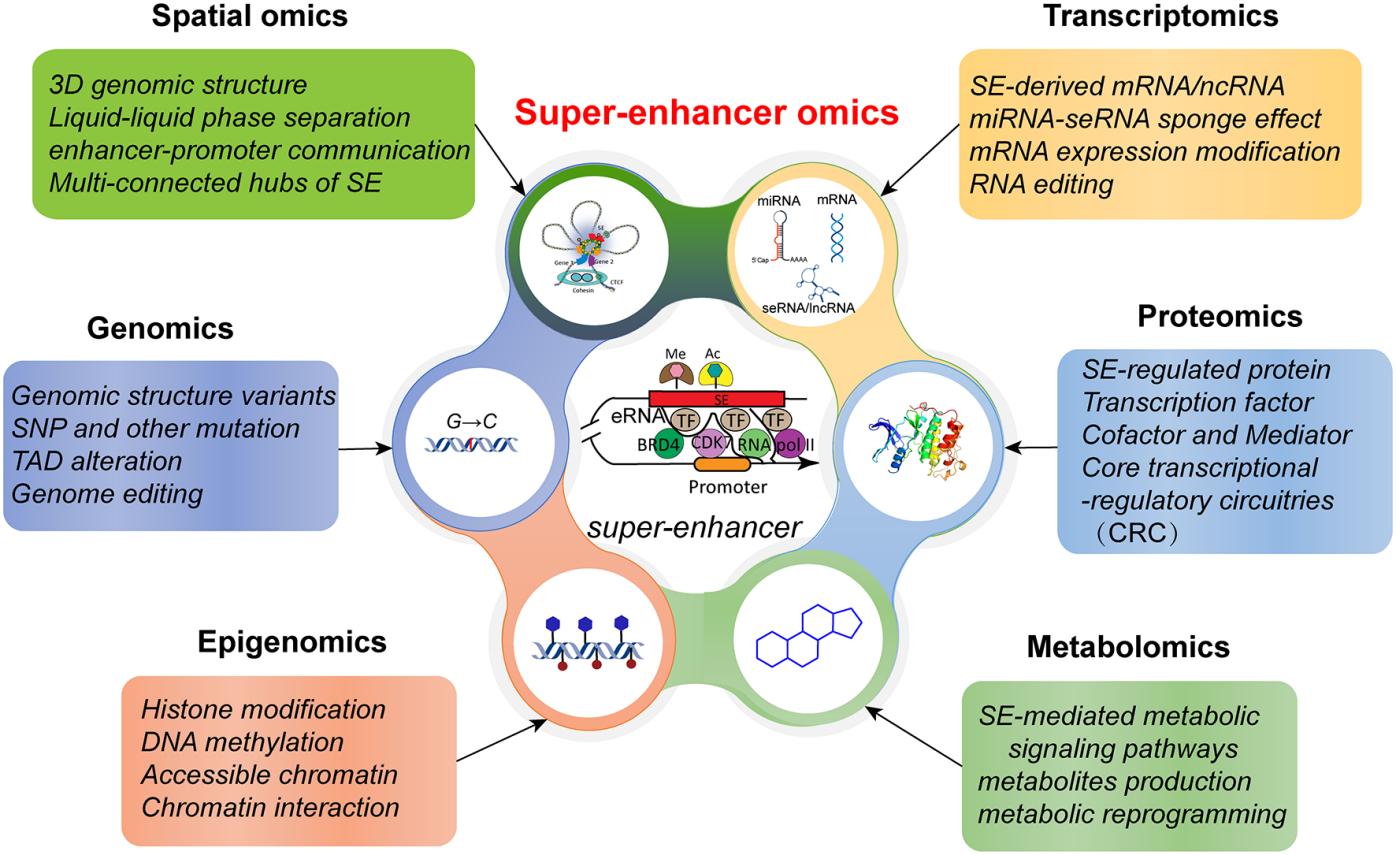
Overview of the Super-enhancer omics related with Pan-omics

Super-enhancers (SEs) consist of multiple stitched enhancers that are denser on the chromatin, forming a longer, more intense enhancer clusters than typical enhancers (TEs) [6]. A super transcriptional regulation complex or platform is consists of Mediators, RNA polymerase II and super-enhancer bound with very high amounts of TFs [7] (Fig. 1). The spatial, complexity and dynamic 3D architecture and functions of SEs have unveiled the emergence of “super-enhancer omics”. Super-enhancer omics is integrated and inextricably interacted with the “Pan-omics” through the major roles of transcriptional regulation (Fig. 1). SEs facilitate the transcriptional regulation on mRNA and other noncoding RNA, such as eRNA, seRNA and lncRNA. Epigenomics modification, such as Histone modification, accessible chromatin interaction, DNA methylation probably changes spatiotemporal organization of SEs. Genomics alterations have the potential to remodel the structure and function of SE, thereby affecting the transcriptomics. SEs also navigate the transcriptional expression of master TFs and downstream target genes through “core transcriptional regulatory circuitry” (CRC), and then direct proteomics alterations involved in diverse significant signaling pathways, such as stem cell characteristics. Metabolic abnormalities of the malignant phenotype of tumor cells are strongly associated with SE-mediated glucose, lipid and animo acid reprogramming [8].

Dysregulation of SE-mediated transcriptional machinery can disrupt stem cell features, such as processes of self-renewal [9], reprogramming [10], and lineage differentiation of ESCs and induced pluripotent stem cells (iPSCs) [11]. Attractively, pharmacological inhibitors targeting critical components of SE assembly and their activation have shown great promise in stem cell reprogramming and growth inhibition of cancer stem cells in several pre-clinical models.

The importance of SEs in transcriptional control involved in various diseases, such as cancers [12–14], immunology disorders [7, 15], cardiovascular disease [16], have been well documented. In this review, we highlight the multifaceted regulatory roles of SEs in determining stem cell features through interacting with Pan-omics, which will facilitate the development of stem cell-based therapeutics in the future.

## Prediction and identification of super-enhancer

**Fig. 2:**
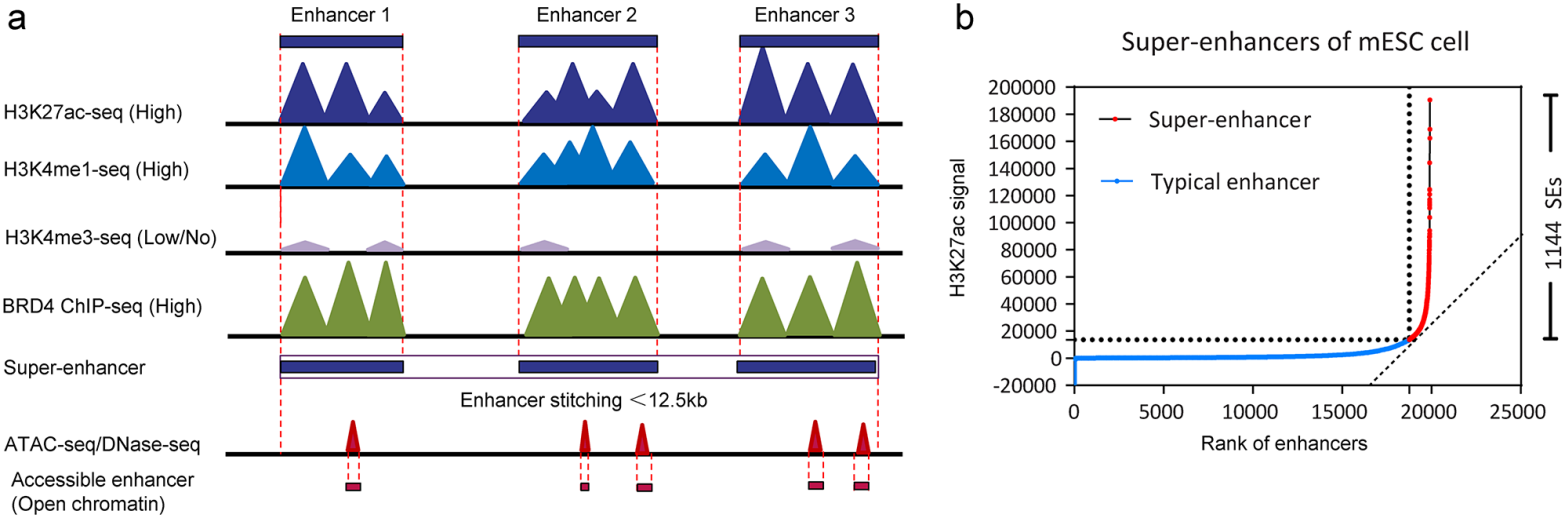
Prediction and identification of super-enhancer. **(a)** High throughput sequencing methods are used to detect signal of enhancer and active enhancers. **(b)** The slope of ranking of enhancers to separate super-enahncers and typical enhancers. ChIP-seq signal of H3K27ac was obtained from mESC cell on SEdb database

To predict and identify enhancers located in the genome, high-throughput sequencing methods (Table 1), such as ChIP-seq [17] and Fixed-tissue ChIP-seq (FiTAc-seq) [18] have been employed to identify active enhancers enriched in high signal of H3K27ac, high H3K4me1 and low/no H3K4me3 [19] (Fig. 2a). This level of physical contact between regulatory elements such as promoters, enhancers, insulators, silencers, etc., and chromatin DNA is referred to as chromatin accessibility. The methods include Assay for targeting accessible-chromatin with sequencing (ATAC-seq) [20, 21], DNase-seq [22], micrococcal nuclease sequencing (MNase-seq) [23], formaldehyde-assisted isolation of regulatory elements sequencing (FAIRE-seq) [24] have been used to detect open chromatin, on which active enhancers always located. Other derivative sequencing methods can detect the interaction of enhancers and other regulatory elements, such as chromosome conformation capture (3 C) [25], circular chromosome conformation capture (4 C) [26] and chromosome conformation capture carbon copy (5 C) [27], ChIP followed by selective isolation of chromatin-associated proteins (ChIP-SICAP) [28], Cleavage Under Targets and Tagmentation (CUT&Tag) [29], Chromatin Interaction Analysis by Paired-End Tag sequencing (ChIA-PET) [30]. Some sequencing methods are further used to reveal eRNAs and reverse inference enhancer position, such as GRO-seq [31] and STARR-seq [32].These sequencing techniques have provided insights into the spatial, complexity, and dynamic characteristics of SEs in different cell types and diseases [33–38].

**Table 1:** High-throughput sequencing involved in super-enhancer omics

Name	Methods	Function related to SE
Assay for targeting accessible-chromatin with sequencing (ATAC-seq)	The Tn5 transposase, preloaded with DNA adapters, is used to simultaneously cut the genome and label the cut DNA fragments. Due to steric hindrance, most of the DNA tag sequences are integrated into open chromatin regions.	To detect open or protected areas of chromatin.
Chromatin immunoprecipitation sequencing (ChIP-seq)	Using antibodies to immunoprecipitate DNA-binding proteins associated with chromatin complexes, and then sequencing to obtain the DNA sequence.	Histone markers and master TFs binding signal to identify SE.
ChIP followed by selective isolation of chromatin-associated proteins (ChIP-SICAP)	Useing antibodies to precipitate protein-DNA complexes, and then identifies the specific interaction sites and regions between these proteins and DNA through mass spectrometry analysis.	This technique enables high-throughput, comprehensive analysis of protein-enhancer interactions.
Chromosome conformation capture (3 C)	Chemical cross-linking fixes chromatin and connecting regions that are close to each other in the chromatin by enzymatic cleavage, then by using PCR to detect these connections.	Revealing the spatial distances and interactions between different regions on the chromosome.
Circular chromosome conformation capture (4 C)	Studying the interactions of a specific DNA sequence with the entire genome, rather than being limited to single interaction analysis.	Detecting the interactions between SE and other regions of the genome.
Chromosome conformation capture carbon copy (5 C)	Detecting interactions between multiple DNA sequence segments, and providing broader coverage data chromosome spatial structure and gene regulatory networks.	Providing comprehensive, higher resolution on interactions between enhancers and other genomic regions.
Cleavage Under Targets and Tagmentation (CUT&Tag)	By using specific antibodies to target transposase near DNA-binding proteins, introducing DNA molecular tags at this location, and then determining the binding position of DNA-binding proteins through sequencing.	Identifying the Interaction positions between DNA-binding proteins and enhancer on the genome.
DNase-seq	DNase I enzyme digests cleave DNA sequences located in open chromatin regions, without cleaving DNA sequences in densely packed chromatin regions. By sequencing the DNA sequences cleaved by the enzyme, open chromatin regions can be identified.	Identifying enhancer sequences located in open chromatin regions.
Formaldehyde-assisted isolation of reg-ulatory elements sequencing (FAIRE-seq)	Using formaldehyde treatment to separate open chromatin regions from densely packed regions, and then performs sequencing analysis on these open regions.	Identifying enhancer sequences located in open chromatin regions.
Fixed-tissue ChIP-seq (FiTAc-seq)	A modified protocol that replaces the proteinase K digestion applied in FiT-seq with extended heating at 65 °C in a higher concentration of detergent and a minimized sonication step, to produce robust genome-wide H3K27ac maps.	To detect SE using formalin-fixed, paraffin-embedded (FFPE) tissues.
Global Run-On Sequencing (GRO-Seq)	A Nuclear Run-On assay coupled to deep sequencing to assess real-time transcription from engaged RNA polymerase.	A high-resolution map of annotation and quantification of short-lived RNA molecules, such as eRNA.
High-throughput/resolution chromosome conformation capture (Hi-C)	A high-throughput sequencing-based chromatin interaction analysis approach that operates genome-wide without the constraint of specific interacting proteins, using a “all vs. all” mode.	Revealing the spatial configuration and interaction between enhancers and nearby gene loci in the three-dimensional
Chromatin Interaction Analysis by Paired-End Tag sequencing (ChIA-PET)	Using chromatin immunoprecipitation (ChIP) to enrich interacting chromatin regions, followed by linking these interacting chromatin regions using an adapter ligation method, and finally conducting sequencing.	To determine the interaction relationships between enhancers and nearby gene loci, regulatory elements, or other chromatin regions.
Self-Transcribing Active Regulatory Region–seq (STARR-seq)	Utilizing transcriptionally active DNA regions to transcribe into RNA, and then sequenced to determine the DNA sequences with regulatory functions.	Directly measuring the transcriptional activity of regulatory regions to identify enhancers and other regulatory elements.

A computerized algorithm called “Rank Ordering of Super-Enhancers” (ROSE) was developed to predict SEs based on ChIP-seq signal in 2013 [6]. This algorithm stitches together enhancers located within a certain distance (<12.5 kb) and ranks all stitched enhancers based on their signal strengths (Fig. 2a). The slope of the sorted signal intensity curve is tangent with the 45° diagonal line (Fig. 2b). The tangential position is used to determine the threshold signal to distinguish SEs and TEs [6, 39]. The signal strength of SEs was higher than TEs, which was accessible to disperse via the cut-off value. The SE curves of stem cell line mESC were plotted using the SE marker of H3K27ac (Fig. 2b).

Several tools and databases have been developed to analyze SEs in different cell types (Table 2). The user-friendly integrated software tool NaivSE automates the processing of raw sequencing data into a comprehensive annotated report of predicted SEs [40]. Interactive databases, such as dbSUPER [41], SEdb [42], SELER [43], and SEanalysis [44] also provide the integrating analysis for SE, SNP, long non-coding RNA, master TF from various cell types.

**Table 2:** Super-enhancer associated databases

Database	Functions of database	Website	Ref.
TRlnc	Provides the detailed (epi) genetic information in transcriptional regulatory regions (promoter, enhancer/super-enhancer and chromatin accessibility regions) of lncRNAs.	http://bio.licpathway.net/TRlnc	[202]
SEA version 3.0	Provides a comprehensive database of all available SE information across multiple species.	http://sea.edbc.org	[203]
SEanalysis	Provides a comprehensive SE regulatory analysis, involving the identification of SE-associated genes, TFs occupying these SEs and the upstream signaling pathways of identified TFs.	http://licpathway.net/SEanalysis	[204]
SELER	A novel database integrating large amounts of experimental and computational data to decode the regulatory functions of SE-lncRNAs in tumorigenesis.	http://www.seler.cn	[43]
SEdb	Provides detailed genetic and epigenetic annotation information on human super-enhancers.	http://www.licpathway.net/sedb	[205]
SEdb 2.0	SEdb 2.0: a comprehensive super-enhancer database of human and mouse	http://www.licpathway.net/sedb	[206]
TRCirc	Documents TF-circRNA regulatory relationships, provides other regulatory information about transcription of circRNAs, including SE- associated circRNAs.	http://www.licpathway.net/TRCirc	[207]
SKmDB	Provide preliminary data analyses including next generation sequencing, gene/isoform expression levels, gene co-expression subnetworks, as well as assembly of putative lincRNAs, typical and super enhancers and transcription factor hotspots available in the human and mouse skeletal muscle tissues and cells.	http://sunlab.cpy.cuhk.edu.hk/SKmDB	[208]
dbCoRC	The first comprehensive and interactive database of core transcription regulatory circuitry based on the mapping of SE and prediction of TF binding sites.	http://dbcorc.cam-su.org	[209]
SEA	Integrates super-enhancers in multiple species and annotates their roles in the regulation of cell identity gene expression.	http://sea.edbc.org	[210]
dbSUPER	The first integrated and interactive database of super-enhancers, which provides transcriptional regulation resources of cell identity and disease.	http://bioinfo.au.tsinghua.edu.cn/dbsuper/	[41]
KnockTF	Provides details about TFs binding to promoters, SEs and typical enhancers of target genes.	http://www.licpathway.net/KnockTF/index.html	[211]
Cistrome Cancer	A comprehensive resource for predicted TF targets, enhancer profiles and SE target genes in cancers.	http://cistrome.org/CistromeCancer/	[212]
DEEPSEN	A method for genome-wide prediction of super-enhancers based on convolutional neural networks	https://github.com/1991Troy/DEEPSEN	[213]
ATACdb	Provides detailed (epi) genetic annotations in chromatin accessibility regions, including super-enhancers, typical enhancers, TFs.	http://www.licpathway.net/ATACdb	[214]
VARAdb	A useful resource for selecting potential functional variations including SE.	http://www.licpathway.net/VARAdb/	[215]
NaviSE	A user-friendly streamlined solution for SE analysis, annotation and navigation.	https://sourceforge.net/projects/navise-superenhancer/	[40]

## Biological characteristics of super-enhancer omics

### Spatial structure and function unit of SEs

**Fig. 3:**
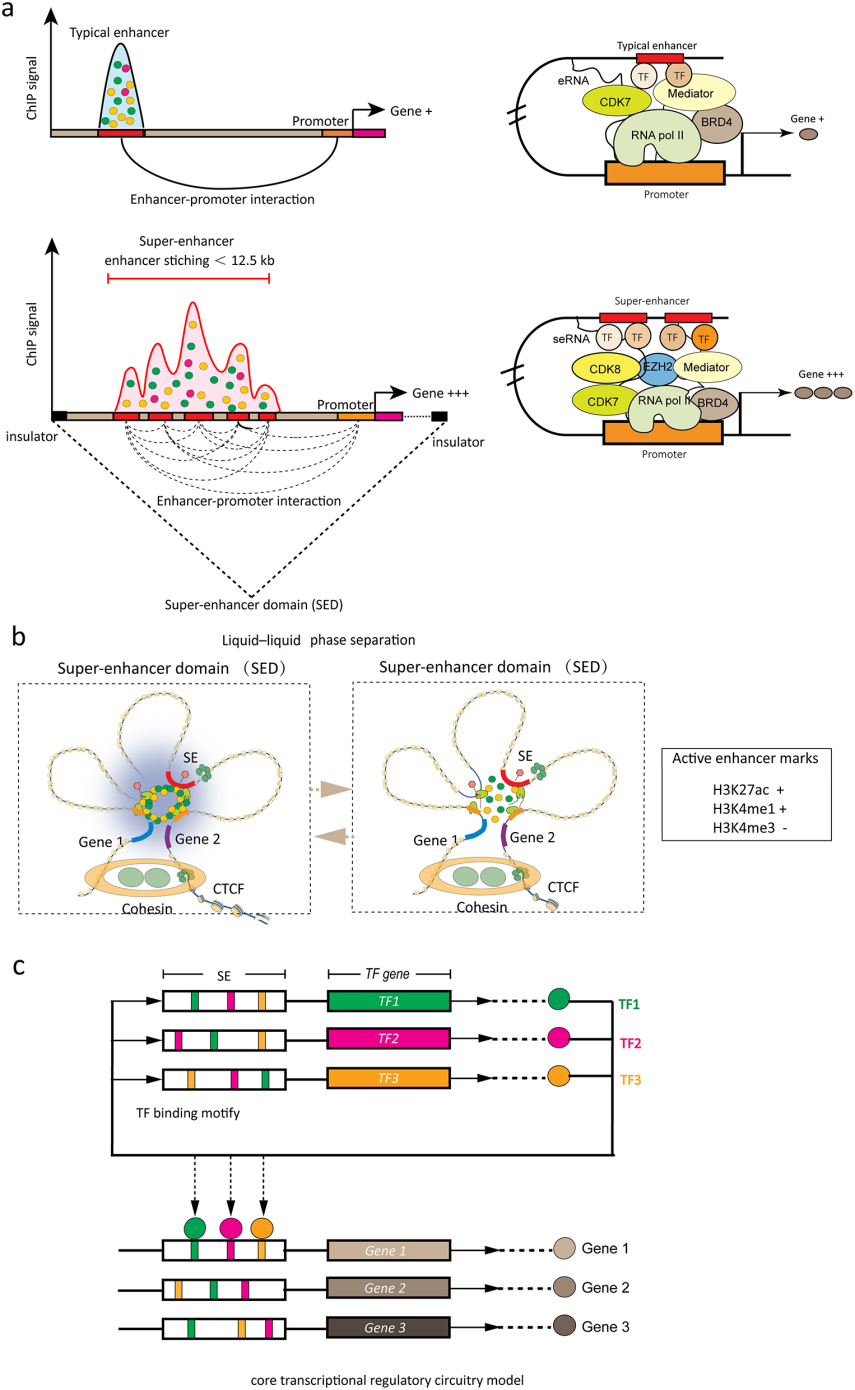
Illustration of cartoon graphics about super-enhancer (SE) and super-enhancer domain (SED). **(a)** Schematic depiction of the classic model of cooperativity for typical enhancers and super-enhancers. The higher density of transcriptional regulators, including transcriptional factors, Mediators, and activators through cooperative binding to SEs contributes to higher transcriptional output, higher enhancer-promoter interactions and increased sensitivity to regulators concentration at SEs than typical enhancers. Super-enhancer acts as a signal integration platform controlling tissue-specific gene expression within the super-enhancer domain (SED). **(b)** Structure of super-enhancer and super-enhancer domain (SED) in the liquid-liquid phase separation model. Liquid-liquid phase separation model illustrates dynamic SE activation. Many molecules bound at SEs, can undergo reversible chemical modifications (e.g., acetylation, phosphorylation) at multiple sites, interacting with multiple other components, thus forming ‘‘crosslinks’’. This cross-link was a reversible feature that can be reversibly modified, or any other feature involved in dynamic binding and unbinding interactions. **(c)** Super-enhancers drive core transcriptional regulatory circuitries. Master TFs self-regulate via inward binding to their cis-binding element within SE, regulating a coordinated set of TFs. The interconnected auto-regulatory loop is termed as “core transcriptional regulatory circuitry” (CRC)

Most of time, SE and SE-driven genes are generally located within topologically associating domains (TAD) formed by the looping of two interacting CTCF sites (CCCTC binding site) co-occupied by cohesion, which is called the super-enhancer domain (SED) (Fig. 3a and b) [45]. The genome structure of eukaryotes is highly organized and hierarchical. DNA and histones assemble into primary chromatin structure, nucleosome and then folded into primary unit TAD, a region of highly interacting chromatin compartmentalized in the metazoan genome [46]. Cohesin facilitates the formation of secondary “gene loops” within TAD, such as cohesin-associated enhancer-promoter loops and cohesin-associated CTCF loops [19, 47]. TADs are demarcated by boundaries, which are regions of less interaction and conserved across cell types and species [47]. The cohesin ChIA-PET data of ESCs revealed that 84% SEs and associated genes were located in the CTCF loop, while only 48% of TEs were within the CTCF loop [45]. SEs and SED possess numerous specific structural and functional characteristics compared to TEs (Fig. 3). Some stitched enhancers within a SED physically interact with each other more frequently than sequences outside the SED and TAD (Fig. 3a) [48]. SED typically contains one SE that loops to its driven gene within the SED [45, 49], appearing to confine SE activity to the gene within the SED [45].

There is one-sidedness in explaining the structure and function of SEs from the 2D perspectives of “SE-regulated gene loop”. Recently, phase-separated multi-molecular assemblies with Hi-C-sequencing further provided a general regulatory mechanism underlying SE formation, function, and properties [49], which explained how two gene promoters exhibit synchronous bursting when activated by the same SE [49] (Fig. 3b). Mediators and co-activators BRD4 and MED1 form phase-separated droplets at SEs that compartmentalize and concentrate the transcription apparatus, suggesting a significant role for co-activators in this process and revealing the regulation mechanisms of function and structure of SE involved in the control of hub cell identity genes [11, 50–53] (Fig. 3b). Live-cell super-resolution and multi-color 3D-imaging approaches investigate putative roles of endogenous condensates in the regulation of SE-controlled *Sox2* [54]. In contrast to enhancer distance, the condensate’s positional dynamics are a better predictor of gene expression of *Sox2* [54].

### Constitute enhancers within SEs

SE are characterized by their significantly longer length and stronger signals [55], composed by multiple constitute enhancers with different functions. The different activities of luciferase reporter vectors carrying different fragments of the constitute enhancer indicated that the transaction activity of constitute enhancers within SEs were all kinds of difference [6]. Moreover, superimposing or synergistic effects on increasing gene expression by these constitute enhancers were not observed, indicating that the interplay among the constitute enhancers has more complicated mechanisms, even the counterproductive impact [56]. To further clarify the endogenous functions of constitute enhancer within SE, classical enhancers and facilitator elements were found in α-globin-SE [57]. Facilitators lack intrinsic enhancer activity, yet their absence impairs classical enhancers ability to fully upregulate target genes. Without facilitators, classical enhancers show reduced Mediator recruitment, enhancer RNA transcription, and enhancer-promoter interactions. Facilitators are interchangeable but exhibit functional hierarchy based on their position within a multipartite enhancer. It is worth mentioning that diverse SE regulates a single target gene in different cell types [58]. Transcriptional dysregulation of the *Myc* oncogene in various aggressive tumor cells is generally accomplished by the acquisition of diverse cancer-specific SEs, differing in size and location, interact with the *Myc* gene through a common and conserved CTCF binding site located 2 kb upstream of the *MYC* promoter [59–61].

## Transcriptional regulatory model of SEs

Master TFs, a kind of pioneer TF which self-regulate own expression via recognizing cis-binding element within SE, navigate the expression of a coordinated and correlated set of TFs. The interconnected auto-regulatory loop is termed as “core transcriptional regulatory circuitry” (CRC) (Fig. 3c) [62]. The transcriptional programs that define the identity of cell type and lineage-specific are controlled by master TFs that bind cell-type-specific enhancers, as well as signaling factors, which bring extracellular stimuli to these enhancers [6, 63–65]. Mechanistically, these master TFs also control the acetylation status of the TF binding motifs by recruiting acetylation writers, readers, and erasers, thereby creating and restoring SE [64–66]. Interestingly, dynamic compartmentalization of TFs and coactivators by phase-separated condensates regulates the assembly of transcriptional machinery at genomic loci [52]. Stem cell-associated SEs are pertinent to specific signaling pathways upon which stem cells depend. Most importantly, the dynamically remodel of SEs in stem cells is always the response to the microenvironment changes. For example, in the hair follicle model, dynamical remodeling of SE rapidly and effectively maintained homeostasis or responded to damage repair in the hair follicle stem cell [67]. Thus, CRC models draw the landscape of master TF forming the CRC regulation pattern and prove valuable for further investigating the roles of cell-type-specific master TF on transcriptional regulation in healthy and diseased cells [65].

### Alterations of TAD remodel SE and gene expressions

**Fig. 4:**
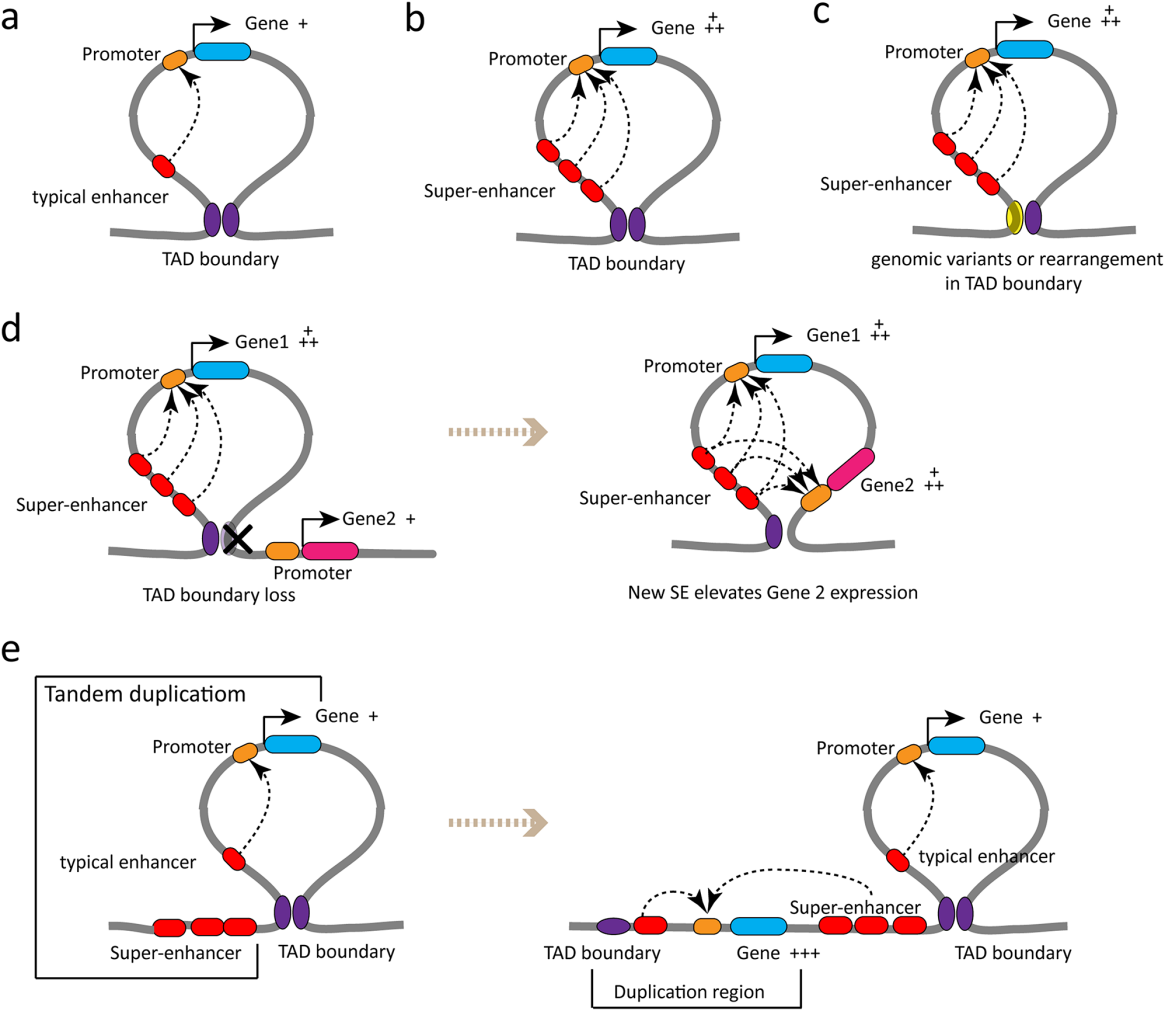
Alterations of TAD boundary remodel SE and gene expressions. **(a)** Typical enhancer (TE) regulates downstream gene within TAD. **(b)** SEs extensively upregulate the expression of a driven gene within TAD. **(c)** Genomic variants or rearrangement in TAD boundary alter 3D chromatin architecture leading to the dysregulation of the driven gene. **(d)** TAD boundary loss alters 3D chromatin architecture and aberrant SE-promoter interactions and increases the expression of other neighboring gene. **(e)** Model for high-level gene overexpression at the *IGF2* locus in CRC, which involves TanDup-mediated de novo contact domain formation resulting in the hijacking of a lineage-specific SE

TAD boundary defines the typical and SE-regulated genes (Fig. 4a and b), but the alterations of TAD boundary always lead to SE dysregulation on nearest genes. The disruption of TAD boundaries by genomic structural variants or rearrangement affects the expression of nearby genes and provides growth advantages to certain diseases [68–70] (Fig. 4c). Loss of TAD boundary results in dysregulation of SE and aberrant activation of nearby genes previously located outside that boundary [45, 71] (Fig. 4d). Deleting *Prdm14* gene TAD boundaries of two CTCF sites caused a 4.5-fold increase in *Slco5a1* expression, located next to *Prdm14* [72]. SEs insulated by strong TAD boundaries may act as a functional unit to promote oncogenesis in cancer cells [50, 71]. Recurrent tandem duplications intersecting with a TAD boundary mediate de novo formation of a 3D contact domain comprising IGF2 and a lineage-specific SE, resulting in high-level activation of IGF2 [73] (Fig. 4e). These findings imply that the implementation of SEs function is first determined by the correct structure of SED and TAD. Once the spatial structure of SED and TAD is disturbed and reshaped, SE is vulnerable to dysregulate nearest genes outside the SED.

### SE-derived noncoding RNA transcriptomics

**Fig. 5:**
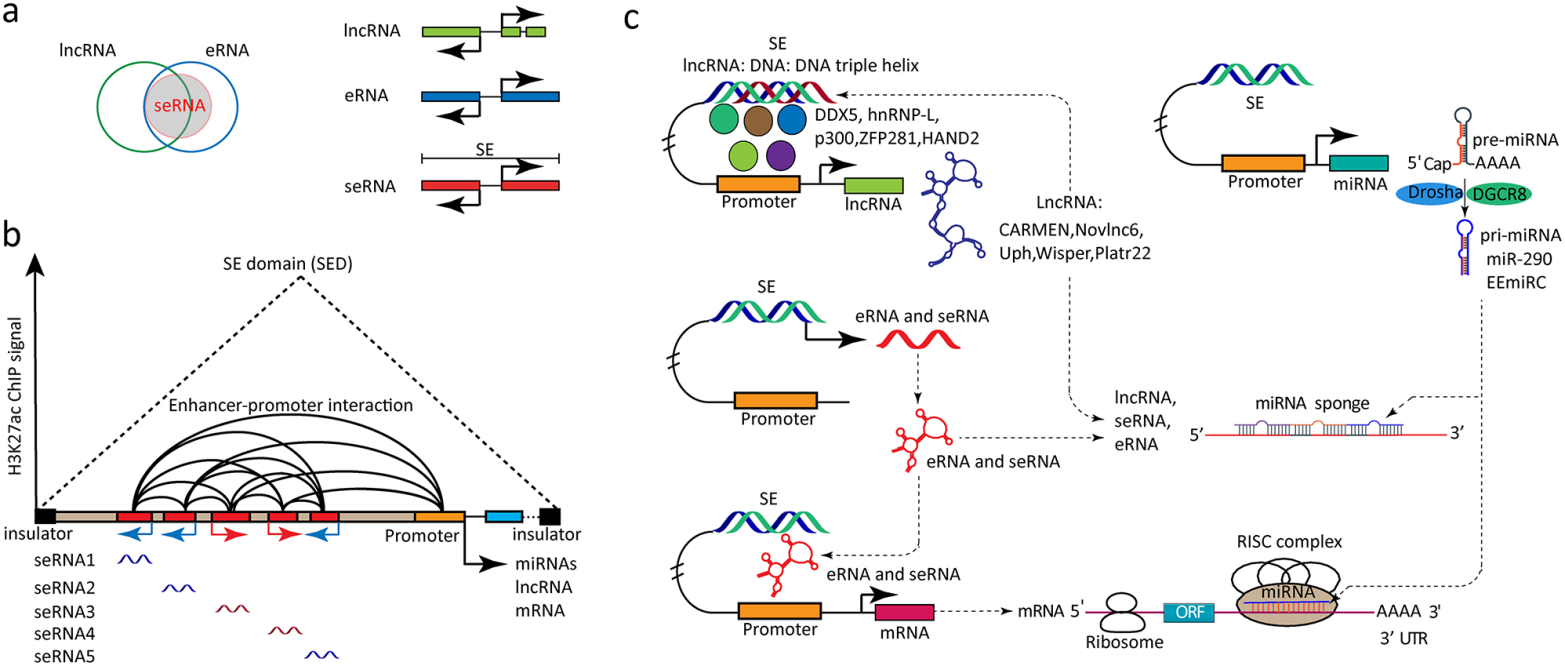
SE-derived ncRNAs. **(a)** The relationship of seRNA, eRNA and lncRNA in Venn plot. LncRNA is usually characterized by its length of more than 200 bp and multiple exon cleavage. eRNAs are mainly defined by the transcription region at enhancers. Some eRNAs are mainly synthesized from SE region, named super-enhancer RNAs (seRNAs). **(b)** Schematic depiction of seRNA transcripted in SE. Most of seRNAs are expressed at SE regions, and a correlation between loss or gain of SE formation and seRNA expression is observed. Many seRNA-producing enhancers within SE were coincident with the active marker H3K27ac. These eRNA transcripts are often bidirectional with low copy numbers, lacking a polyA tail. seRNAs function in facilitating nucleosome depletion, establishing DNA accessibility and enhancer-promoter interactions directly or indirectly to regulate gene expression. **(c)** Mechanisms of SEs govern ncRNA expressions. These ncRNAs mainly include miRNAs, lncRNAs, seRNAs which are widely expressed in various stem cells to form a competitive network to regulate SE activation and the expressions of downstream genes. SE-related lncRNA forms RNA: DNA: DNA triple helix within SED, recruits regulatory factors to SED. SE-related lncRNA influences chromosome structure, acting as a spatial amplifier to promote tissue-specific gene expression related to SE. SEs launch the biogenesis of master miRNAs essential for cell identity by enhancing both transcription and Drosha/DGCR8-mediated primary miRNA (pri-miRNA) processing

Currently, due to the advancement of high-throughput sequencing and analysis techniques, an increasing number of SE-derived ncRNAs have been detected in stem cells (Table 3). These ncRNAs mainly include miRNAs, lncRNAs, seRNAs [74–78], which are widely expressed in various stem cells to form a competitive endogenous RNA network to regulate SE activation and the expressions of downstream genes (Fig. 5). The relationship of seRNA, eRNA and lncRNA are showed in Venn figure (Fig. 5a).

**Table 3:** The SE-derived noncoding RNA

Name	ncRNAs	Effects	Cells	Ref.
CARMEN	lncRNA	regulator of cardiac cell differentiation and homeostasis	Cardiac cell	[79]
Novlnc6	lncRNA	heart development and potential regeneration	Heart cell	[80]
Uph	lncRNA	Uph activates HAND2 expression to facilitate heart development	Heart cell	[81]
Wisper	lncRNA	Cell identity and regulate the gene expression program	Cardiac fibroblasts	[82]
Platr22	lncRNA	Pluripotency maintenance and proper differentiation	mESCs	[83]
*Nanog-eRNA*	seRNA	Sustain pluripotency via seRNA producing by these SEs	ESCs	[99]
Stage-specific seRNAs	seRNA	myofibril assembly and heart development	ESC	[100]
RUNX1-eR1	eRNA	regulate RUNX1 expression	HSCs and hemogenic endothelial cells	[101]
EEmiRC	miRNA	a novel enhancer within the pluripotency associated microRNA cluster	E14.1 ES cells	[102]
pri-miRNA and master miRNA	miRNA	SEs launch the biogenesis of master miRNAs essential for cell identity by enhancing both transcription and Drosha/DGCR8-mediated primary miRNA (pri-miRNA) processing.	ESCs, Pro-B cells, myotubes	[74]
mir290-SEs	miR290	DNA methylation at the mir290-SEs on the cellular differentiation state	mESCs	[103]

LncRNA is usually characterized by its length of more than 200 bp and multiple exon cleavage. SE-related lncRNA, a kind of lncRNA that forms RNA: DNA: DNA triple helix within SED, recruits regulatory factors to SED. SE-related lncRNA influences chromosome structure, acting as a spatial amplifier to promote tissue-specific gene expression related to SE. Cardiomyocyte-specific SE-derived lncRNA, CARMEN, is an essential regulator of cardiac cell differentiation and homeostasis [79]. A SE-derived lncRNA, Novlnc6, is involved in heart development and potential regeneration [80]. A SE-derived lncRNA Uph interacts with HAND2 and activates its transcription to facilitate heart development [81]. SE-derived lncRNA Wisper, crucial for stem cell identity, can regulate the gene expression program of cardiac fibroblasts [82]. SE-derived lncRNA Platr22 transcripts coat chromatin near the SE region and interact with DDX5, hnRNP-L and p300 to active transcription of nearby pluripotency regulator ZFP281, contributing to pluripotency maintenance and proper differentiation of mESCs [83]. These findings propose that these TFs, Mediators and lncRNA assemble into a transcription complex, thus promoting an open and active epigenetic chromatin state, sustaining stem cell features.

Enhancer RNA (eRNA) is a new subfamily of unspliced ncRNAs synthesized at the enhancer region. eRNAs are short and unspliced with lower intracellular stability and shorter half-life. Some eRNAs are similar to lncRNA with long, cleaved sequences or poly-A tail, which are more stable. Some eRNAs are mainly synthesized from the SE region, named super-enhancer RNAs (seRNAs), specifically related to maintaining stem cell pluripotency and features of cancer cells [84–87]. LncRNA is usually characterized by its length more than 200 bp and multiple exon cleavage, while eRNA is mainly defined by its transcription region. Compared with lncRNA, most of eRNA are short and unspliced with lower intracellular stability and shorter half-life. There are also some eRNA structures similar to lncRNA with long, cleaved sequence or poly-A tail, which are more stability [88].

eRNAs function in facilitating nucleosome depletion, establishing DNA accessibility and enhancer-promoter interactions directly or indirectly to regulate gene expression by transcription pre-initiation complexes RNA pol II, DNA- and RNA-binding transcription factor, or co-factor of DNA- and RNA-binding transcription factor [89]. eRNAs bind to adhesin and contribute to the dynamic stability of the enhancer-promoter cycle in cis-acting and regulate chromatin remodeling events in trans-acting [90]. eRNAs promote loop formation of enhancers and promoters, transcription repressors, and recruit transcription activators to activate promoters [91].

Sequence-specific transcription factors bind to enhances to promote the coordinated combination of chromatin-remodeling enzymes, histone-modifying enzymes, cofactors, and finally RNA polymerase II complex (RNA pol II) [84, 88, 92, 93]. The early transcription termination of eRNAs may be regulated by the integron complex in a manner that relies on the termination to trigger polyadenylation (PaA)-like signal [94]. These eRNA transcripts are often bidirectional with low copy numbers, lacking a polyA tail [95]. Depleting these eRNAs leads to the decreased expression of their neighboring protein-coding genes, including master regulators of cellular differentiation [96]. However, not all active enhancers can be transcribed to produce eRNA. Similar to eRNA, most of seRNAs are expressed at SE regions, and a correlation between loss or gain of SE formation and seRNA expression is observed [85, 86, 97]. The transcription of enhancers within genes interfered with and attenuated host gene transcription during productive elongation, but the behavior of enhancer-transcription (rather than eRNAs themselves) explained this attenuation [98]. RNA exosome protected divergently transcribed lncRNA expressing enhancers by resolving deleterious transcription-coupled secondary DNA structures, also regulating long-range SE chromosomal interactions important for ESCs function [85].

Schematic depiction of seRNA transcription is showed in Fig. 5b. Many seRNA-producing enhancers within SE were coincident with the active mark H3K27ac, decreased DNA methylation, and enrichment for the DNA hydroxylase Tet1 in ESCs [84]. The remote SEs at the *Nanog* locus differentially regulated adjacent genes related to pluripotency via seRNA producing by these SEs in ESCs [99]. In ESCs, 95 and 78 seRNAs associated with early- and late-stage ESC differentiation were screed out, respectively [100]. The binding sites of master regulators of ESC differentiation, including NANOG, FOXA2, and MYC, were significantly observed in the loci of the stage-specific seRNAs [100]. Interestingly, the RUNX1 intronic enhancer (eR1) transcribes from the RUNX1-SE and acts in *cis* to regulate RUNX1 expression in HSCs and hemogenic endothelial cells [101]. In the future, studies that uncover the mechanisms that control enhancer transcription and eRNA and seRNA function will improve our understanding of gene regulation and help us understand stem cell.

MicroRNAs (miRNAs) are approximately 22-nucleotide-long, small non-coding RNAs that post-transcriptionally regulate gene expression. A 332-bp intragenic enhancer activates the transcription of the early embryonic microRNA cluster (EEmiRC) locus in mice, presumably through binding of transcription modulators like OCT3/4, SOX2, and CTCF [102]. Deleting the intragenic enhancer significantly decreased the transcription of the EEmiRC, further proving that this region dominates the expression of EEmiRC [102].SEs launch the biogenesis of master miRNAs essential for cell identity by enhancing both transcription and Drosha/DGCR8-mediated primary miRNA (pri-miRNA) processing [74]. The methylation of enhancer is closely related to the heterogeneity of cells, and functionally affects the transcription and cell state of mESCs [103]. DNA methylation at the Sox2 and the mir290-SEs is independently regulated and has distinct consequences on the cellular differentiation state [103]. Thus, the mechanisms of SE governing ncRNA expressions are displayed in Fig. 5c.

## Super-enhancer omics in stem cell

### SEs determine stem cell identity via self-renewal and pluripotency

**Fig. 6:**
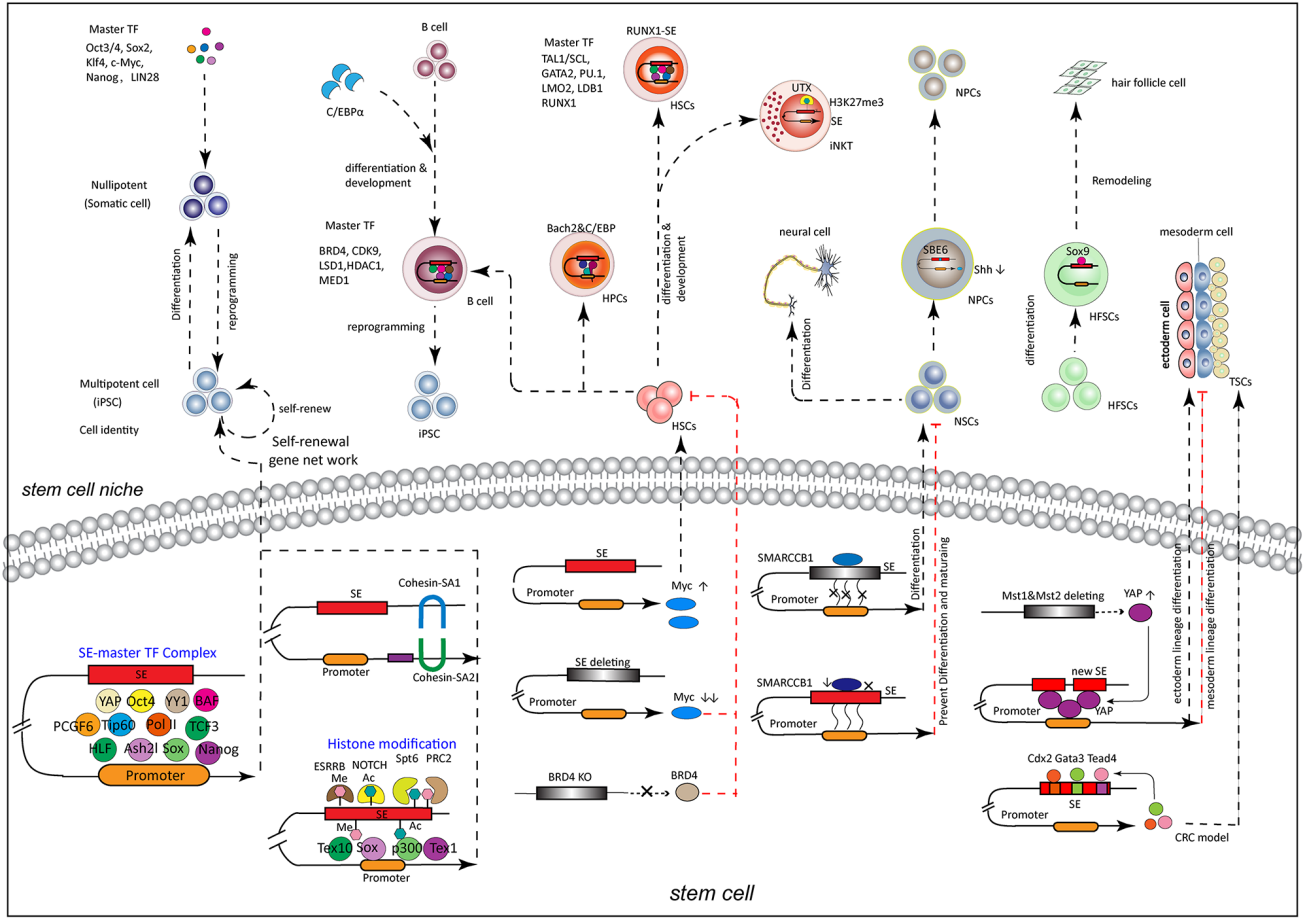
Super-enhancer omics in stem cell. SEs determine stem cell identity via stem cell self-renewal/pluripotency and govern stem cell reprogramming, remodeling, differentiation and development

Self-renewal is a crucial way to perpetuate stem cells by controlling their division to produce more stem cells under an undifferentiated state, to restore the stem cell pool after an injury and stimulus, and to maintain stem characteristics in the adult tissues [104, 105]. In this procedure, stem cells maintain pluripotency, an ability to differentiate into various cell types [106]. The ability of self-renewal is promoted in stem cells, but the potential of differentiation is restricted. ESCs were firstly isolated from the inner cell mass of blastocysts in 1981, often used in stem cell-related characteristics and markers [107]. The multiple regulatory roles of SEs on the self-renewal of stem cells are showed in Fig. 6.

Pluripotency and self-renew-related factors always form SE-complex to up-regulate stemness genes and maintain self-renew and pluripotency ability in stem cells. Typical pluripotency master TFs OCT4, SOX2, and NANOG bind SEs and recruit Mediator BRD4 to activate stemness-related genes to sustain pluripotent ESCs [6, 108]. Pluripotency-associated SEs (SE-SOX2, SE-PIM1, and SE-FGFR1) displayed remarkable conservation in placental mammals and were sufficient to drive reporter gene expression in a pluripotency-dependent manner. Disruption of these conserved SEs through the CRISPR-Cas9 approach severely impaired stem cell pluripotency.

Ash2l formed an enhancer-bound Ash2l/OCT4/SOX2/NANOG complex that can drive enhancer activation, govern pluripotency network, and stemness circuitry [9]. The complex of YY1 and BAF can up-regulate the proliferation and pluripotency of mESCs by interacting with OCT4, and stimulate transcription by enriching at the SEs region [109]. PCGF6 activates pluripotency-related genes by forming a complex with OCT4 and regulating interactions of SEs and promoters [110]. Tip60 plays as a co-activator of Pol II promoters accompanying SEs, and governs the c-Myc network in ESCs, thereby potentiating self-renewal and cell metabolism [111]. Chimeric transcription factor TCF3-HLF recruits HLF binding sites at hematopoietic stem cell/myeloid lineage-associated SEs to drive lineage identity and self-renewal [112]. Transcriptional factor NFIB and NFIX as crucial rheostats of tissue homeostasis govern SE maintenance of the critical hair follicle resident stem cells -specific TF genes, which direct the lineage cell fate choice [113].

Many master TF and coactivators remodel histone acetylation and methylation status within the SE region to restore the self-renewal and pluripotency of stem cells. Tex10, a key pluripotency factor, enriched on SEs by interacting with SOX2, Tet1 and p300, regulates SEs acetylation and DNA demethylation in self-renewal, pluripotency and reprogramming of ESCs and embryo development [10]. ESRRB has a regulatory effect on CpG methylation dynamics enhancer units of ESCs-specific SEs. The silencing of ESRRB leads to selective SEs inactivation and the reduction of pluripotency [114]. Two variants of Cohesin-SA1 and Cohesin-SA2 jointly control the spatial structure of SEs in mESCs. Interestingly, the two variants maintain the stability of SEs through opposite actions, thereby regulating the self-renewal and cell identity of stem cells [115]. SEs feed-forward loops amplify the signal level of the NOTCH TF family, mediate the rapid transition between two semi-stable states of neuroblastoma-adrenergic and mesenchymal [116]. This transition also mediates the remodeling of histone acetylation and changes in the SEs landscape [116]. Spt6 enriches at SEs to control the acetylation and methylation of H3K27 and seRNA transcription by competing with PRC2 methyltransferase in ESCs [117, 118]. Deletion of Spt6 reduces pluripotency factors expression [117, 118]. Thus, the master transcription regulators of core pluripotency circuitry in stem cell, binding with chromatin-modifying enzymes, signal transduction components, proximal and distal regulatory elements including typical enhancers and SEs, promote the self-renewal ability by occupying genes regulating stem cell ground state [119, 120].

### SEs govern stem cell reprogramming and remodeling

Reprogramming refers to how cells change epigenetic expression and restore pluripotency, a process of “de-differentiation” [121]. The reprogramming from somatic cells to iPSCs develops a new method to obtain patient-derived PSCs facing fewer ethical issues. There are many explanations for the mechanism of reprogramming, such as the “Elite model”, “Stochastic and deterministic”, “two-step process” and “reversal of development” [122]. By transferring the critical factors in ESCs into somatic cells, Yamanaka, et al., successfully reprogrammed the iPSCs [123]. OCT3/4, SOX2, KLF4, c-Myc, NANOG and LIN2, were identified as the essential and sufficient factors to generate iPSCs in later research [124]. In addition to the mentioned reprogramming factors, there are also reprogramming activators that increase the reprogramming efficiency, including pluripotency-associated genes, cell cycle-regulating genes, and epigenetic modifiers [122].

SEs differ from TEs in size, transcription factor density and content, ability to activate transcription and sensitivity to perturbation (Fig. 6). Therefore, it is much easier to remodel stem cells after the specific targeting to master TF and SE-associated coactivators and downstream genes involved in self-renewal, reprogramming, and pluripotency. Exposing B cells to C/EBPα pulses can increase the abundance of complex proteins related to SEs, including BRD4, CDK9, LSD1, HDAC1, and MED1, thereby inducing the reprogramming of B cells to iPSCs [125].

Under the balance of self-renewal in niches, adult stem cells keep their lineage selection and progression during tissue homeostasis but often display fate flexibility outside their niche [67]. The global landscape of SEs in hair follicle stem cells was mapped in their native niche. SEs and their dense clusters (‘epicentres’) of Sox9 binding sites underlie the identity, lineage commitment and remodeling of adult stem cells in vivo [67]. Furthermore, the new fate of stem cells was acquired by decommissioning old and establishing new SEs and/or epicenters, an auto-regulatory process that abates one master regulator subset while enhancing another [67].

### SEs regulate stem cell differentiation and development

Stem cells are a type of under differentiated precursor cells with multidirectional differentiation and development capabilities. In recent years, the multi-directional differentiation potential and molecular mechanism of stem cells have made rapid progress, which provides new strategies for the regeneration and repair of biomedical engineering tissues [126]. Moreover, the continuous and rapid development of stem cell therapy has gradually entered the clinical stage, thereby changing the current situation of medical care [127]. Nevertheless, a significant difficulty with stem cell therapy is to control its differentiation through desired cells or tissues.

Master TFs and Mediators involved in critical signaling pathways always regulate stem cell differentiation and development via SE (Fig. 6). Hippo/Yap signaling pathway is a crossroad converging on stem cell development, which is involved in lineage differentiation by regulating the formation of YAP-bound SEs as the key transcriptional regulatory circuit of ESC [11, 128]. After Hippo kinase Mst1 and Mst2 are depleted in mESCs, the upregulation of YAP and nuclear translocation lead to the formation of new SEs, which promote the expression of genes that drive ectoderm lineage differentiation and inhibit mesoderm lineage differentiation [11]. A large number of SE-predicted trophoblast stem cells (TSC)-specific trophectoderm-specific TFs show dynamic expression patterns during the differentiation of TSC [129]. Five SE-TFs (FOS, GATA2, MAFK, TEAD4, and TFAP2C) have a proclivity for regulating each other, and constituting a gene regulatory network to control the placental gene expression program in TSCs [130].

Hematopoietic stem cells (HSCs) in the bone marrow have long-term self-renewal ability and the potential to differentiate into various types of mature blood cells, rebuilding the human hematopoietic system and immune system [131]. BRD4 conditional knockout (KO) mice analysis showed that BRD4 was required for hematopoietic stem cell expansion and progenitor development [132]. GATA factors mediate transcriptional changes through a stage-specific interplay with regulatory elements: GATA1 binds different sets of regulatory elements in erythroid progenitors and precursors and controls the transcription of distinct genes during commitment and differentiation [133]. An evolutionarily conserved region of SE located 1.7 megabases downstream of *Myc* is essential for regulating its expression in normal HSCs and leukemic stem cell (LSCs) hierarchies in mice and humans [134]. Deleting this SE in mice leads to a complete loss of c-Myc expression in HSCs and progenitors, contributing to an accumulation of differentiation-arrested multipotent progenitors and loss of myeloid and B cells, mimicking the phenotype caused by Mx1-Cre-mediated conditional deletion of the Myc gene in HSCs [134]. RUNX1 and other hematopoietic TFs, TAL1/SCL, GATA2, PU.1, LMO2 and LDB1 bind at RUNX1-SE, which is observed in normal HSCs and T-cell acute lymphoblastic leukemia cells, regulate HSCs differentiation and development [101]. Bach1 and Bach2 are transcription inhibitors, which can inhibit genes essential for myeloid cells and myeloid genes to promote the development of B cells in common lymphoid progenitor cells [135]. The Bach2 and C/EBP families share target genes in hematopoietic progenitor cells, but they oppose each other and regulate the SEs in myeloid genes [136]. In the process of erythroid commitment and differentiation, H3K27 acetylation is gradually lost, and the use of activity enhancers and SEs is reduced [133]. H3K27me3 histone demethylase UTX regulating the accessibility of SEs promotes lineage-specific gene expression and the development of Invariant natural killer T (iNKT), innate-like lymphocytes [137]. All the above studies have shown that master TF, Mediators and other histone acetylation and methylation alterations affect HSCs differentiation via SE modification. In-depth research on the differentiation mechanism of HSCs is expected to bring new hopes for the clinical treatment of blood diseases.

Neural stem cells (NSCs), a life-long source of neurons and glia, can self-renew and differentiate into nerve cells, which is crucial in the development of the central nervous system [138]. However, using human somatic cell-derived NSCs and their progeny to improve neurological diseases is still facing many problems. The tumor suppressor SMARCCB1, essential for the silencing of SEs in hESC under neural differentiation conditions, inhibits the bivalent genes in hESCs and antagonizes the chromatin accessibility of SEs [139]. Reducing the levels of SMARCB1 prevents stem cells from maturing into brain cells, but not other types of cells [139]. Sonic hedgehog gene (Shh) brain enhancer 6 (SBE6) is necessary for the proper expression of Shh in neural progenitor cells (NPC) and is active in vertebrate brain and neural tube development [140]. During the differentiation of ESCs into neural progenitor cells, the spatial proximity of the enhancer-promoter of Shh was reduced [141]. After the differentiation of mouse ESCs into NSCs, the intensity of chromatin loops increased, and participating in the developmental program triggers the establishment of chromatin conformational states. The pre-existing structure is enhanced in more mature cell types, and the newly formed loop domain contains enhancers that are activated during differentiation [142].

### Super-enhancers maintain hallmarks of cancer stem cell

**Fig. 7:**
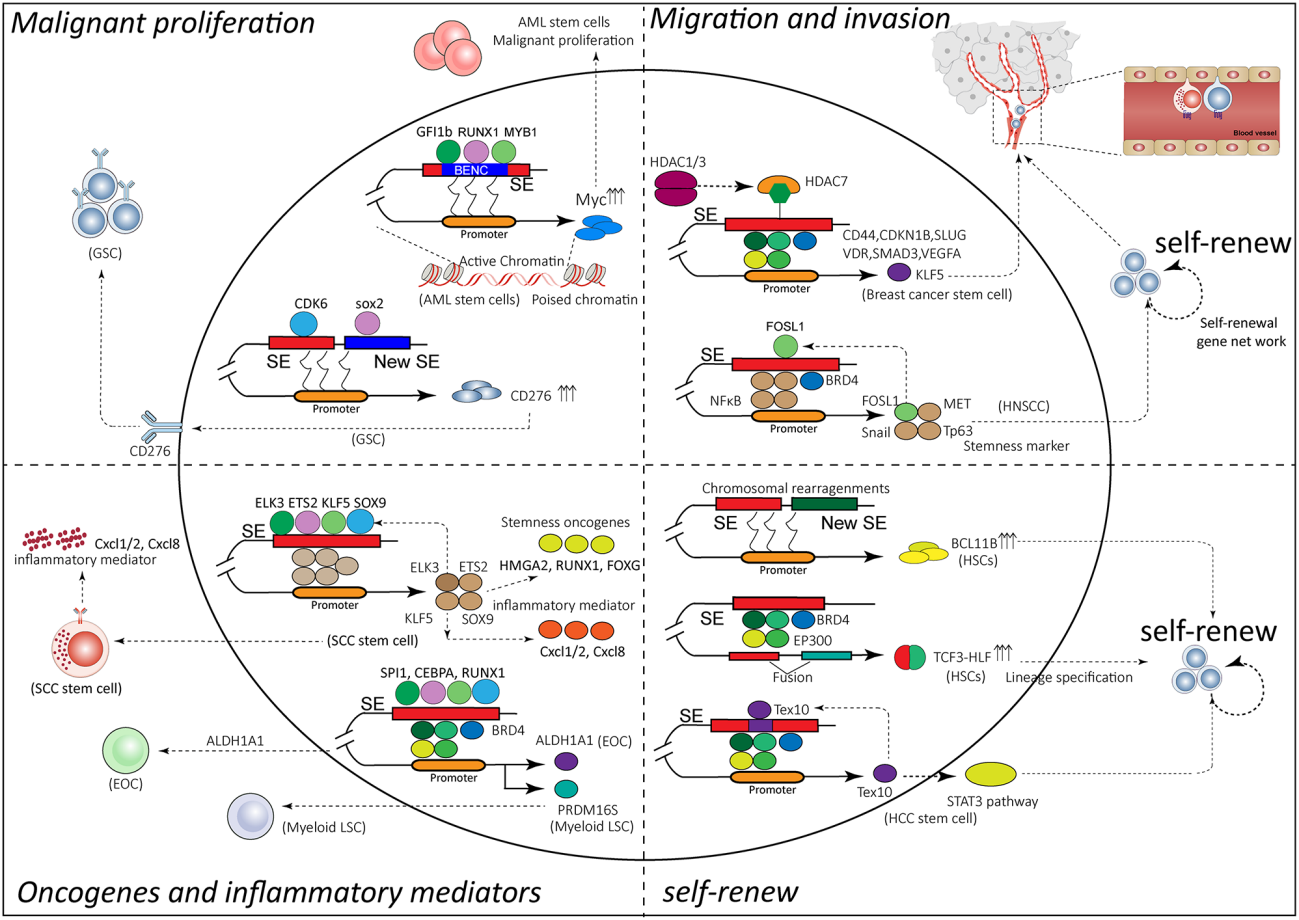
Super-enhancer maintains the hallmarks of cancer stem cells. SE maintains the oncogenic, malignant proliferation stemness hallmarks of CSCs via master transcriptional factors and mediators, forming a CRC model to explosively upregulate their expression levels in a positive feedback manner. Genomic mutation and epigenetic alterations also hijack SEs to trigger cancer stem cells

Cancer stem cells (CSCs), a small subset of cells with self-renewal, differentiation and highly tumorigenic ability, are closely related to the occurrence, development, recurrence and treatment resistance in tumors [143]. CSCs characteristics attribute to the acquisition of gene mutations, epigenetic shift, and alterations of the multicellular microenvironment. SEs in embryonic stem cells are sometimes hijacked to fuel the self-renew of cancer stem cells [144], leading to cancer relapse and drug resistance [145, 146]. The emerging role of SEs on various cancers has been well documented in previous reviews [14, 55, 87, 147], but the functions in CSC are not fully covered. This review provides the current understanding of mechanisms that SE maintains the oncogenic and stemness hallmarks of CSCs (Fig. 7), showing promise to eradicate the CSCs and future challenges on the path to cure.

### Stemness and oncogenic SEs modulate cancer stem cells

Considering the essential roles of master TFs, Mediators, and other hub regulators on stem cells via SE-navigated complex, the malignant features of CSCs are determined by stemness-SEs and specific oncogenic SEs. Numerous master TF and mediators form a CRC model to explosively upregulate their expression levels in a positive feedback manner to maintain stemness in CSCs. A ‘blood enhancer cluster’ (BENC) of SE comprises multiple enhancer modules with selective activity, recruiting a compendium of TFs, including GFI1b, RUNX1 and MYB, and precisely controlling the Myc levels throughout most of the hematopoietic hierarchy [134]. BENC exhibits increased chromatin accessibility in human acute myeloid leukemia stem cells compared to blasts [134]. BENC may form highly combinatorial systems that allow precise control of gene expression across normal cellular hierarchies and can be hijacked in malignancies.

Given the malignant roles of leukemia stem cells (LSCs) in chronic myelogenous leukemia (CML) relapse of patients, the precise regulation of LSC stemness is further needed. X-box binding protein 1 (XBP1), known as a substrate of mRNA-splicing endonuclease IRE1α in the unfolded protein response pathway, was a SE-associated oncogene in LSCs [148]. XBP1-SE supported survival and self-renewal capacity in primary CML CD34^+^ cells, but eradicated LSCs in CML mice and impaired the leukemogenesis of LSCs in CML mice after the knockdown of XBP1 [148].

Testis expressed 10 (Tex10), a new core component of the pluripotency circuitry, positively promoted ESC self-renewal through SE [10]. Tex10 is overexpressed in poorly-differentiated HCC cells and increased the number of stem cell spheroids in the self-renewal test, promoting liver cancer stemness via STAT3 pathways [149]. SE-associated gene *FOSL1* promotes tumorigenicity and metastasis in head and neck squamous cell carcinoma (HNSCC) by upregulating cancer stemness and pro-metastatic genes, including Snail2 and FOSL1 itself [150]. Furthermore, BRD4 recruits Mediators and NF-κB p65 to form SEs at *TP63*, *MET*, *FOSL1*, and triggers transcription of cancer stemness genes and pro-metastatic genes in HNSCC [151]. Disrupting SEs by BRD4 inhibitors potently suppressed self-renewal, invasive growth, lymph node metastasis of CSC, and at last eliminated CSCs from human HNSCC [151].

Compared to normal skin stem cells, SEs regulate the expression of a series of specific stem-related oncogenes in skin squamous cell carcinoma (SCC) stem cells, such as HMGA2, RUNX1 and FOXG [152]. In addition, master stemness TF, including ELK3, ETS2, KLF5, and SOX9, bind to SE regions to regulate their expressions and essential stem cell fate genes, thereby forming a CRC model to maintain the stem cell properties [152, 153]. ETS2-SE transcriptional regulated ETS2 and inflammatory mediators, *Cxcl1/2* and *Cxcl8*, and affected SCC stem cell growth [152]. An active chromatin landscape was mapped across 44 patient-derived glioblastoma stem cells (GSCs), 50 primary tumors, and 10 neural stem cells (NSCs) using the multi-omics datasets, including gene expression, whole exomes, copy number profiles, and DNA methylation [154]. The essential SE-associated genes and the core TFs established by SEs and maintaining GSC identity were illustrated [154]. Core glioblastoma stem cells (GSC)-associated genes (such as CDK6 and SOX2) were up-regulated by recurrent SEs, thereby maintaining GSC properties and promoting the malignancy of glioblastoma [154]. Consistently, the formation of new SE-promoter interaction regulated the expression of stemness-related CD276 in GSC [155]. Oncogenic TF KLF5 was correlated with the stemness of breast cancer cells [156]. A SE located downstream of the *KLF5* gene positively regulated itself in basal-like breast cancers (BLBC), promoting the stem-like characteristics of breast cancer cells [157]. In epithelial ovarian cancer (EOC), BRD4-mediated SEs regulate the expression of a series of stem-related genes, including a significant cancer stem cell marker ALDH1A1 [158]. In embryonal tumors with multilayered rosettes (ETMRs), the SE-dependent oncogenic C19MC-LIN28A-MYCN circuit contributes to embryonic epigenetic programming and stem cell maintenance [159].

### Genomic and epigenetic alterations hijack SEs in cancer stem cell

Recently, genomic mutation and epigenetic alterations also hijack SEs to trigger cancer stem cells. The chimeric TCF3-HLF defines an incurable ALL subtype [112, 160]. TCF3-HLF fusions hijack endogenous stem cell functions to drive leukemia by activating SE, which are generally active in hematopoietic stem and progenitor cells (HSPCs) [112]. Moreover, SE activity of TCF3-HLF is mediated through the recruitment of EP300; thus, BRD4 inhibitor JQ1 and EP300 inhibitor A-485 are two reasonable options for TCF3-HLF-fusion leukemia [112]. Aberrant allele-specific deregulations of BCL11B co-opted into a gene regulatory network that drives transformation by maintaining the state of lineage ambiguous leukemia stem cells [161, 162]. Chromosomal rearrangements that juxtapose BCL11B to SEs active in hematopoietic progenitors, or focal amplifications, generating a SE distal to BCL11B, drive human lineage ambiguous HSC as an oncogenic actuator [161]. *H1F0* codes linker histone H1.0, whose expression is low in pluripotent cells but high in somatic cells [163]. The differential methylation status of SE region within a CpG island (CGI) shore governs the diverse expressions of *H1F0* in many tumors [164, 165]. The silencing of the *H1F0* due to epigenetic change is helpful for the maintenance and self-renewal of CSCs [164, 165]. Histone deacetylases HDAC1 and HDAC7 are necessary to maintain CSCs in both breast and ovarian tumors [166, 167]. HDAC1/3 only induces the activation of HDAC7 in breast CSCs, but not in non-stem tumor cells [166]. HDAC7 enhances H3K27ac signaling at stem-like transcription factors, such as *CD44*, *CDKN1B*, *SLUG*, *VDR*, *SMAD3*, and *VEGFA*, by binding to their SEs [166]. Targeting the HDAC1/3-HDAC7 axis (such as MS-275) may contribute to the inhibition of the CSCs phenotype.

## Targeting Super-enhancers components

**Fig. 8:**
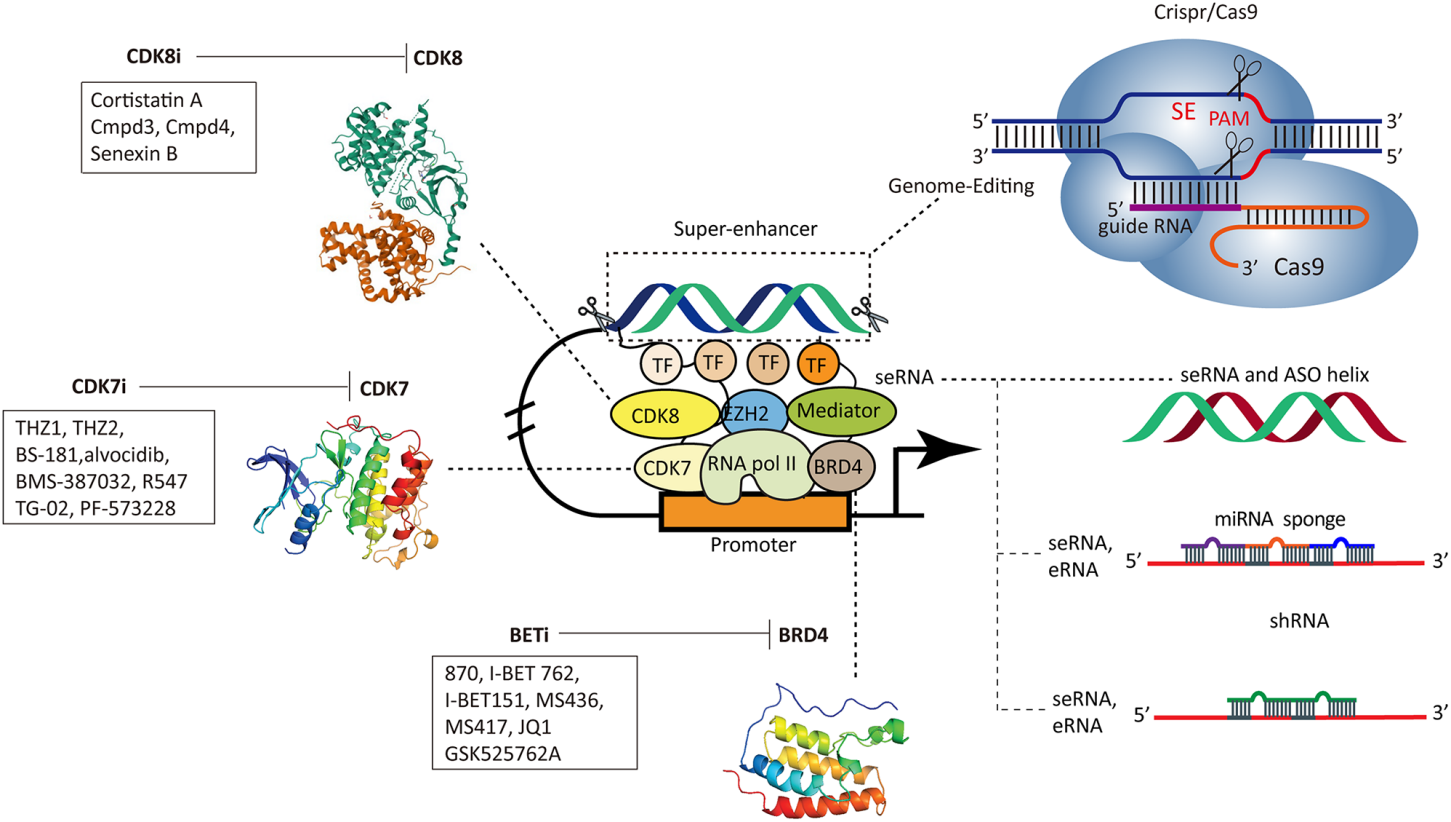
Targeting SE components. BRD4, CDK7, CDK8 and other SE components were specifically inhibited by small molecular inhibitors. Antisense oligonucleotides (ASOs), shRNA, miRNA and CRISPR/Cas9 genome editing system also interrupt seRNA and eRNA or target gene expression

### Small molecular inhibitors against SE components

BRD4, as a general transcriptional regulator, recruits SE-mediated transcriptional regulatory complexes via recognizing histone acetylated chromatin sites [168, 169]. BRD4 is considered to be a pharmacological inhibition target showing therapeutic activity in various diseases. The widely-used BRD4 inhibitor JQ1 significantly decreased populations of CSCs in vitro and in vivo by targeting the BRD4-mediated transcriptions at SEs in head and neck cancer (HNSCC) [170]. Moreover, JQ1 showed an inhibitory effect on iPS reprogramming [125] and ESC multi-differentiation [108]. Particularly, JQ1 alternated cancer stem cell features, such as the stemness of basal-like breast cancer [157] and stem cell marker ALDH activity in ovarian cancer [158]. Interestingly, BET inhibitors inhibit ALDH activity by eliminating BRD4-mediated ALDH1A1 expression through the activation of SEs and related eRNAs [158], offering a theoretical basis for the clinical application of JQ1 to inhibit the growth of cisplatin-treated ovarian cancer cells in vitro and in vivo. Other BETi, including compound 870, I-BET 762, I-BET151, MS436, MS417, GSK525762A, have also demonstrated inhibitory effects on the stemness of basal-like breast cancer [157], ALDH activity [158], reprogramming of acute myeloid leukemia stem cells [171] and ESC multi-differentiation [108]. JQ1 reduced the activity of ALDH by destroying SE elements and down-regulating the stem-related target genes of BRD4. Clinically, the combination of JQ1 and cisplatin were beneficial for improving drug sensitivity and survival outcomes [158] (Fig. 8).

Cyclin-dependent kinase 7 (CDK7), the catalytic subunit of the Cdk-activating kinase (CAK) complex, has been implicated in the control of cell cycle progression and of RNA polymerase II (RNA pol II)-mediated transcription in the stem cell [172, 173]. The CDK7/9 inhibitor SNS-032 significantly suppressed cellular proliferation and induced apoptosis [174]. It also inhibited the outgrowth of xenografted uveal melanoma cells and patient-derived xenograft (PDX) tumors in NOD-SCID mice and repressed the cancer stem-like cell (CSLC) properties of uveal melanoma through transcriptional inhibition of stemness-related protein Krüppel-like factor 4 (KLF4) [174]. THZ1, a well-known CDK7 inhibitor [175], suppressed the expression of stem-related gene KLF5 by inhibiting SE activity in BLBC [157]. Blockages of the SE-associated gene transcription by THZ1 exterminated LSCs in retroviral BCR-ABL–driven CML mice while sparing normal hematopoietic stem cells [148]. Many other inhibitors of CDK7, such as THZ1, THZ2, BS-181, alvocidib, BMS-387,032, R547, TG-02, PF-573,228, were found to abolish cancer cell, probably specific targeted against CSC selectively [176] (Fig. 8).

Interestingly, compared to CDK7 and BRD4, the inhibition of CDK8/19 has an opposite mechanism which enhances mediator activity and the recruitment of mediator to RNA Pol II, and upregulate enhancers and SEs activities thus stabilizing the naïve pluripotency of hESCs [177]. The natural product cortistatin A (CA) selectively inhibited mediator-associated CDK8 and CDK19, and has anti-leukaemic activity in vitro and in vivo, then disproportionately induces upregulation of SE-associated tumor suppressor and lineage-controlling functions, including the TFs CEBPA, IRF8, IRF1, and ETV6 [178, 179] in CA-sensitive AML cell lines but not in CA-insensitive cell lines [180].

Enhancer of zeste homolog 2 (EZH2), the catalytic subunit of Polycomb repressor complex 2, is highly expressed in cancer stem cells of numerous malignant tumors and has a critical function in cancer stem cell expansion and maintenance [181] (Fig. 8). Inhibition of EZH2 stabilized the SEs and maintained the cell identity and pluripotency of human periodontal ligament stem cells [182, 183]. GZ17-6.02 (three synthetic components of GZ17S; curcumin, harmine, and isovanillin) could reverse the carcinogenic effects of SEs and CSC markers by lowering the acetylation of these genes [184]. As for myeloid leukemia, PRDM16S, occupying the SE sites of myeloid master regulators SPI1, CEBPA, and RUNX1, inhibited megakaryocytic/erythroid potential and promoted the maintenance of myeloid LSCs. This fate conversion may be rescued by SPI1 inhibitor in PRDM16S-induced leukemia [185].

### Antisense oligonucleotides (ASOs)

Antisense oligonucleotides (ASOs) are chemically synthesized nucleic acid analogs that have been widely used to disrupt target gene expression via binding to DNA/RNA according to Watson-Crick base pairing [186]. Many findings revealed the therapeutic potential of targeting ncRNA by ASO against cancers in vitro and in vivo [187]. Modified ASOs could precisely down-regulated the levels of seRNA and its nearest neighbor genes *Dppa3* by disrupting the SE-promoter loop [99]. *Wisper* as a cardiac fibroblast-enriched SE-associated lncRNA could be attenuated myocardial infarction (MI)-induced fibrosis and cardiac dysfunction by ASO-mediated silencing in vivo, which represents an attractive therapeutic target to reduce the pathological development of cardiac fibrosis in response to MI and prevent adverse remodeling in the damaged heart [82]. Therapeutic ASO has high specificity and capability of modulating seRNA and enhancer that are not readily druggable. Efficiently delivering of ASO to their targets is still the biggest challenge to overcome in promoting their clinical translations [188] (Fig. 8).

### CRISPR/Cas9 genome editing system

CRISPR/Cas9 system (Clustered Regularly Interspaced Short Palindromic Repeats-CRISPR-associated protein 9) is a groundbreaking gene-editing technology [189]. CRISPR/Cas9 technology directly edits master TFs and SE to investigate the essential roles of SE on stem cells [190]. A features-oriented CRISPR-utilized systematic screen on Oct4-bound CRISPR was performed to screen and identify 16 functional stem-enhancers related to the maintenance of pluripotency [191]. A histone acetyltransferase Kat6b knockout embryonic stem cell line was constructed by CRISPR/Cas9 and found that Kat6b regulates the organization of chromatin and its interaction with pluripotent TF, thereby affecting the neural differentiation and self-renewal of mESCs [192]. After applying compound selectivity engineering and CRISPR/Cas9 genome editing system, the ERK5 signaling pathway maintains ESCs in the naive state and suppresses progression toward primed pluripotency and neuroectoderm differentiation via inhibiting a cardiomyocyte-specific differentiation program [193].

Nowadays, CRISPR/Cas9 deletion systems are also developing to directly delete enhancers in stem cells to study the functions of SE and isolated enhancers [194]. SE acting on common target genes fine-tune the transcription output of their target genes in a partially redundant manner in mESC [194]. The cis-regulatory elements within SE responsible for early replication, compartmentalization, and local genome structure were identified through performing CRISPR/Cas9-mediated deletion and pluripotency-related inversion of replication domains in mESCs [47]. A features-oriented CRISPR-Cas9-utilized systematic (FOCUS) was performed and found 16 Oct4-bound cis-regulatory elements (CREs) within stem cell enhancers important for pluripotency maintenance in mESCs [191]. After the monoallelic and biallelic deletion of the entire 13-kb Sox2-SE in mouse ESCs by a simple and highly efficient double-CRISPR genome editing strategy, Sox2-SE was responsible for over 90% of Sox2 expression and proved the vital roles of Sox2-SE on maintaining the pluripotency of mESC [195]. CRISPR/Cas9-mediated editing-out of RUNX1 enhancer (eR1) within its intragenic SE, or BRD4 depletion by shRNA, repressed RUNX1, inhibited cell growth and induced cell lethality in AML cells expressing mtRUNX1 [196]. CRISPR/Cas9 was used to systematically delete three discrete SE at the *NANOG* locus in ESCs, revealing functional differences in Nanog transcriptional regulation [99]. One distal super-enhancer 45 kb upstream of Nanog (-45 enhancer) regulates both nearest neighbor genes, *Nanog* and *Dppa3*. Interestingly, eRNAs produced at the − 45 enhancer specifically regulate *Dppa3* expression by stabilizing the looping of the − 45 enhancer and Dppa3. Thus, these findings illustrate that genomic editing is required to determine enhancer function and points to a method to target a subset of SE-regulated genes by depleting eRNAs selectively (Fig. 8).

Interestingly, a programmable acetyltransferase based on the CRISPR/Cas9 gene regulation system could effectively activate genes in the enhancer region [197]. Recently, a novel epigenome editing technology (enCRISPRa and enCRISPRi) for the study of enhancer functions has been developed in vivo and in vitro [198]. enCRISPRa and enCRISRPi reshape the epigenetic modification of the target region, thereby activating or inhibiting the transcriptional activity of enhancers and their target genes. The author uses enhancer perturbations of the enCRISPRi mouse model to reveal the lineage-specific requirements of developmental enhancers during hematopoietic lineage differentiation [198]. Therefore, targeting for SE, eRNA, master TFs and other Mediators by small molecular compounds and ASO could restore SE status and interfere eRNA levels, thereby affecting stem cell features (Fig. 8).

## Conclusions, perspectives and challenges ahead

The recent identification of active enhancer landscape in stem cells, together with the discovery that most enhancers and eRNA affect the stemness genes, has generated considerable interest in mapping SE in stem cells. However, challenges remain in identifying the specific SE and associated genes and several vital questions in the field of stem cell remain poorly understood. The skeptics raised doubts that SEs are arbitrarily defined by the algorithm, but there is no functional significance to the cut-off value between super- and typical-enhancers [17]. Therefore, understanding the mechanism of SE switched specific transcription control in stem cell is still our paramount concern.

To understand the complexity of “Super-enhancer omics”, a multi-omics approach is widely utilized in stem cell research in the future, which may answer the hot issues. (i) The function of master TFs and binding motify in SE is needed to be verified. TF binding motify in SE is verified by ChIP-seq, STARR–seq, EMSA and luciferase reporter gene assay in vitro and in vivo. (ii) Methylation patterns of SE genomic sequence are needed to study. Whole-genome bisulfite sequencing (WGBS) reveals the hypo-methylated status in active enhancers and other regulatory elements in SE. (iii) Open chromatin regions of SE in specific stem cell should further be investigated. Accessible regions within SE can be identified by ATAC–seq, DNase-seq and FAIRE–seq, in which the integration sites of exogenous DNA targeted to genomic DNA using an enzyme defining the accessible regions of chromatin. (iv) SE–promoter interactions should be further elucidated. Chromatin conformation capture methods such as ChIA–PET and Hi-C techniques to define distant regions interacting with the same protein. (v) Proteins involved in SE complex are encouraged to screen. ChIP-SICAP and ChIP-MS could identify the hub proteins interacting with SE and master TF, which may be used as the potential drug target. (vi) CRISPR/Cas9 technology is helpful to edit SE. The advent of CRISPR–Cas9 has made mapping the regulatory circuitry of stem cells feasible and creates a minimal targeted deletion to test the activity of specific putative enhancers within SE loci by assessing the consequences of genetic deletions on gene activity.

With the modified sequencing technology are improved, the identification of active or functional enhancers within SE and the interactions among enhancer-promoter based on 3D chromatin conformation are helpful to identity SE in the future [199]. After the conceptual framework of phase separation mode is constructed, it reveals the dynamic changes of hyper-sensitivity SEs responding to a stimulus outside. This model also uncovers the mechanism of SE switch transcriptional control, recruiting clusters of TFs, dynamic changes and simultaneous activation of multiple genes by the same SE. Recently, multi-layered spatial transcriptomics and epigenetics, including single-cell RNA-seq, single-cell ATAC-seq and high throughput chromosome conformation capture technology, also provide an unprecedented and powerful tool for studying SE regulation, stem cell function and fate determination, normal physiology and disease pathogenesis in stem cells [200, 201].

Despite the formidable obstacles ahead, we are currently positioned in a profoundly auspicious and exhilarating era for SE research in stem cells. Our existing expertise, in conjunction with the methodologies expounded above, harbors the potential to eventually comprehend a significant portion of the rationale behind transcriptional regulation mechanisms of SE. This will yield profound implications in the realm of biological investigation, medicine, and biotechnology, advancing our comprehension of spatial and temporal governance over gene expression and empowering the creation of bespoke regulatory elements within SE, possessing tailored potency, inducibility, and resilience, for implementation in medicine.

## Data Availability

No datasets were generated or analysed during the current study.

## Abbreviations

3C: Chromosome conformation capture
3D: Three-dimensional
4C: Circular chromosome conformation capture
5C: Chromosome conformation capture carbon copy
ATAC-seq: Assay for targeting accessible-chromatin with sequencing
BENC: Blood enhancer cluster
BLBC: Basal-like breast cancers
CA: Cortistatin A
CAK: Cdk-activating kinase
CDK7: Cyclin-dependent kinase 7
CGI: CpG island
ChIA-PET: Paired-end tag-seq
ChIP-seq: Chromatin immunoprecipitation sequencing
ChIP-SICAP: Paired-end tag-seq
ChIA-PET: ChIP followed by selective isolation of chromatin-associated proteins
CLL: Chronic lymphocytic leukemia
CML: Chronic lymphocytic leukemia
CRC: Core transcriptional regulatory circuitry
CREs: Cis-regulatory elements
CSLC: Cancer stem-like cell
CUT&Tag: Cleavage Under Targets and Tagmentation
CSCs: Cancer stem cells
EEmiRC: Early embryonic microRNA cluster
EOC: Epithelial ovarian cancer
eRNA: Enhancer RNA
EOC: Epithelial ovarian cancer
ERV: Endogenous retrovirus
ESCs: Embryonic stem cells
ETMRs: Embryonal tumors with multilayered rosettes
FAIRE-seq: Enhancer of zeste homolog 2
EZH2: Formaldehyde-assisted isolation of reg-ulatory elements sequencing
FiTAc-seq: Enhancer of zeste homolog 2
EZH2: Fixed-tissue ChIP-seq
GRO-Seq: Global Run-On Sequencing
GSCs: Glioblastoma stem cells
Hi-C: Global Run-On Sequencing
GRO-Seq: High-throughput/resolution chromosome conformation capture
HNSCC: Head and neck squamous cell carcinoma
HSCs: Hematopoietic stem cells
HSPCs: Hematopoietic stem and progenitor cells
HSCs: Hematopoietic stem cells
iNKT: Invariant natural killer T
iPSCs: Induced pluripotent stem cells
KLF4: Krüppel-like factor 4
LSCs: Leukemic stem cell
LSPCs: Limbal stem/progenitor cells
mESCs: Mouse embryonic stem cells
miRNAs: microRNAs
NPC: Neural progenitor cells
NSCs: Neural stem cells
PDX: Patient-derived xenograft
RNA pol II: RNA polymerase II complex
ROSE: Rank Ordering of Super-Enhancers
SBE6: Sonic hedgehog gene (Shh) brain enhancer
SED: Super-enhancer domain
seRNAs: Super-enhancer RNAs
SEs: Super-enhancers
TAD: Topologically associating domains
TEs: Typical enhancers
Tex10: Testis expressed 10
TFs: Transcription factors
TSC: Trophoblast stem cells
